# Effect of the chemical composition of filter media on the microbial community in wastewater biofilms at different temperatures†
†Electronic supplementary information (ESI) available: Tables S1–S6 are available. See DOI: 10.1039/c6ra21040f



**DOI:** 10.1039/c6ra21040f

**Published:** 2016-10-26

**Authors:** Iffat Naz, Douglas Hodgson, Ann Smith, Julian Marchesi, Safia Ahmed, Claudio Avignone-Rossa, Devendra P. Saroj

**Affiliations:** a Department of Civil and Environmental Engineering, Faculty of Engineering and Physical Sciences, University of Surrey, Guildford GU2 7XH, UK. Email: d.saroj@surrey.ac.uk; b Department of Biology, Qassim University, Buraidah 51452, Kingdom of Saudi Arabia; c Environmental Microbiology Laboratory, Department of Microbiology, Faculty of Biological Sciences, Quaid-i-Azam University, Islamabad, 45320, Pakistan; d Department of Microbial Sciences, University of Surrey, Guildford GU2 7XH, UK; e Cardiff School of Biosciences, Cardiff University, Cardiff CF10 3XQ, UK; f Centre for Digestive and Gut Health, Imperial College London, London W2 1NY, UK

## Abstract

This study investigates the microbial community composition in the biofilms grown on two different support media in fixed biofilm reactors for aerobic wastewater treatment, using next generation sequencing (NGS) technology. The chemical composition of the new type of support medium (TDR) was found to be quite different from the conventionally used support medium (stone). The analysis of 16S rRNA gene fragments recovered from the laboratory scale biofilm system show that biofilm support media and temperature conditions influence bacterial community structure and composition. Greater bacterial diversity was observed under each condition, primarily due to the large number of sequences available and sustenance of rare species. There were 6 phyla found, with the highest relative abundance shown by the phylum *Proteobacteria* (52.71%) followed by *Bacteroidetes* (33.33%), *Actinobacteria* (4.65%), *Firmicutes*, *Verrucomicrobia* (3.1%) and *Chloroflex* (>1%). The dataset showed 17 genera of bacterial populations to be commonly shared under all conditions, suggesting the presence of a core microbial community in the biofilms for wastewater treatment. However, some genera in the biofilms on TDR were observed in high proportions, which may be attributed to its chemical composition, explaining the improved level of wastewater treatment. The findings show that the structure of microbial communities in biofilm systems for wastewater treatment is affected by the properties of support matrix.

## Abstract 2

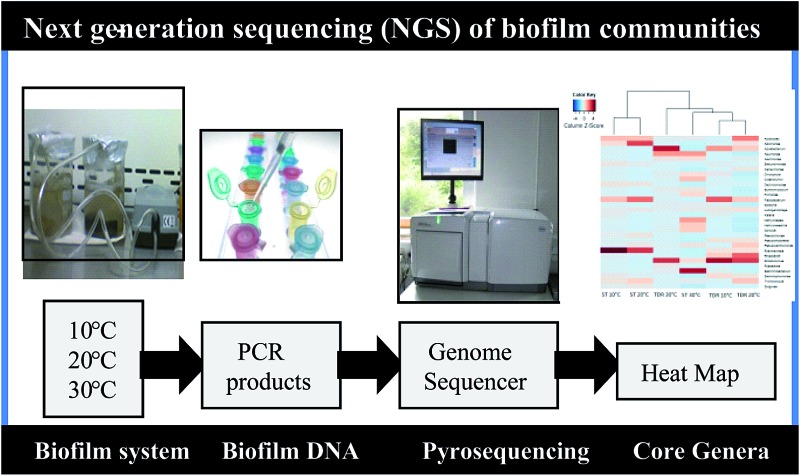
This study investigates the microbial community composition, in the biofilms grown on two different support media in fixed biofilm reactors for aerobic wastewater treatment, using next generation sequencing (NGS) technology.

## 1. Introduction

Biological wastewater treatment systems play an important role in improving water quality and human health worldwide. Harnessing the beneficial activities of naturally occurring microorganisms in bioreactors enables us to remove oxygen-depleting organic contaminants, toxins, and nutrients, while preventing the discharge of pathogens into the environment. The composition, diversity, and dynamics of a microbial community affect the efficiency, robustness, and stability of wastewater treatment systems.^1^ Therefore, the study of the microorganisms in wastewater treatment processes is crucial to better understand the functions and performance of those systems. Moreover, a thorough knowledge of the microbial aspects involved is essential to develop operating strategies and to improve process performance.^2^

Despite the environmental and economic importance of these processes, the knowledge of the microbial communities within biological wastewater treatment systems is limited, primarily because of greater focus on conventional process engineering to achieve immediate goals in practice. Moreover, traditional microbiological techniques and conventional microscopy are insufficient to determine the composition, structure, stability, function and activity of bacteria involved in wastewater treatment processes. For example, culture-dependent methods are biased by the selection of species which do not represent the real dominance structure.^3,4^

However, high-throughput next generation sequencing (NGS) methods provide a more powerful tool for high taxonomic resolution of complex microbial communities.^5^ This can significantly improve the ability to investigate the low-abundance microorganisms.^6^ Recently, NGS technology has been applied successfully in studying microbial communities in the human and animal gut, microbiome, soils, oceans, and various types of bioreactors.^7–12^ Moreover, NGS methods like pyrosequencing was used to characterize bacterial communities from the impellers retrieved from domestic water meters,^13^ natural stream water biofilms,^14^ membrane filtration systems and for metagenomic characterization of environmental microbial communities in wastewater treatment systems.^15–17^ To date, this technique has not been applied to the analysis of bacterial communities of biofilms formed on various packing media used in fixed biofilm reactors (FBRs) for wastewater treatment.

The aim of this study was to provide new knowledge and insight into the structure and composition of bacterial communities found on a commonly used natural support media, stones (ST), and on a synthetic medium, tyre-derived rubber (TDR), during real wastewater treatment. These two materials have a distinct chemical composition; the stone is commonly used in conventional trickling filter systems, the TDR material is not yet used as a support material for full-scale wastewater treatment. The TDR filter media seem to be a suitable option for wastewater treatment because of their ease in availability at low costs and in large quantities. Moreover, TDR media provide a large surface area, high porosity, and resistance to biodegradation.^18^ We sought new understanding of the influence of different temperature conditions and media types on the biofilm composition. To the best of our knowledge, this study represents the first application of NGS to characterize and compare biofilm samples on different types of media used in bioreactors for wastewater treatment. Such information is important for the best operation, transformed engineering design, management of the FBR technology, and the selection of most suitable biofilm support media for wastewater treatment in the areas with higher temperature conditions particularly in the developing countries.

## 2. Experimental section

### 2.1. Evaluation of support media

Two different types of growth supporting media, tyre derived rubber (TDR) and stones/pebbles (ST), were selected to be used as substrates for microbial adhesion. TDR material (discarded bus radial tyre; Michelin, France) were cut into cubical pieces, with each having a surface area of 21.95 cm^2^. Stones (pebbles) with approximately same average surface areas (21.52 cm^2^) were collected from a fresh water stream. X-ray Photoelectron Spectroscopy (XPS) analysis was performed using a Theta Probe Spectrometer (Thermo Fisher Scientific, East Grinstead, UK) for elemental quantification of the surfaces of the selected media. The XPS spectra were acquired using a mono-chromated Al Kα X-ray source (*hν* = 1486.6 eV). The software Avantage (ThermoFisher Scientific, USA) was employed for elemental analysis using the appropriate sensitivity factors and corrections for electron energy analyzer transmission function.

### 2.2. Development of biofilm on media

The biofilm was developed on sterilized media by using biological (wastewater) samples collected from Godalming Sewage Treatment Works, Godalming, UK. Media were incubated in wastewater (300 mL) in bench scale reactors and the biofilm was allowed to develop under aerobic condition, maintained by pumping air at a flow of 4 L min^–1^. All the experiments were conducted in continuous mode, with the addition of freshly collected wastewater for the duration of 14 days in order to observe the changes in biofilm under different temperatures (10, 20 and 30 °C).

### 2.3. Physico-chemical analysis of wastewater

Various physico-chemical factors of the influent and effluent samples were analyzed at three time points during the experimental period of 14 days of biofilm development on both types of packing media. All the analyses were carried out as per APHA.^19^ Each analysis was performed in triplicate for each sample, and the results are expressed as the average.

### 2.4. DNA extraction, PCR amplification and pyrosequencing

Biofilms were removed from both types (ST and TDR) of media developed at 10, 20 and 30 °C in phosphate buffer (PBS) by mechanical action in a vortex.^20^ It was followed by centrifugation at 10 000 × *g* for 5 min to collect the bacterial cells from biofilm pellets. Cell pellets were placed in 100 μL of sterile DNase and RNase free water (Promochem LGC) for DNA isolation.^21^ The DNA was then extracted using a Fast DNA SPIN Kit for Soil (MP Biomedicals).^21^ Quantity and purity of the extracted DNA were assessed in 1.5 μL of the sample using a NanoDrop ND-1000 spectrophotometer (NanoDrop). For the amplification of bacterial 16S rRNA gene fragments, the PCR primers GAGTTTGATCNTGGCTCAG (forward) and GTNTTACNGCGGCKGCTG (reverse) were used. Different barcodes (Table S1†) were incorporated between the 454 adapter and the forward primers to sort each biofilm sample from the mixed pyrosequencing outcomes. Each 50 μL reaction mixture included 1× EF-Taq buffer (Solgent, Daejeon, South Korea), 2.5 units of the EF-Taq polymerase (Solgent), 0.2 mM dNTP mix, 0.1 μM of each primer and 100 ng of template DNA. The PCR profile was as follows: 95 °C for 10 min; 35 cycles at 94 °C for 45 s, 55 °C for 1 min and 72 °C for 1 min, with a final extension at 72 °C for 10 min. The duplicate PCR products were pooled and purified using the QIA quick gel extraction kit (Qiagen, Hilden, Germany), and the purified products were used for pyrosequencing.

### 2.5. Post-run analysis

All partial 16S rRNA gene sequences were preprocessed initially using the pyro-pipeline at the Ribosomal Database Project (RDP) to sort by barcode and remove primers and barcodes from the partial ribotags, and discard low quality and short (<250-bp long) sequences.^22^ These sequences were denoised, assembled into clusters using the precluster command to generate the fasta files datasets (*.fna and *.qual files). These sequences were further analyzed through Mothur.^23^ Chimeras introduced in the PCR process were detected and removed from dataset by using Mothur UChime algorithm.^24^ The processed-sequences were clustered into Operational Taxonomic Units (OTUs) based on 0.97 sequence similarity with the Uclust algorithm.^24^ Representative OTUs were selected based on the most abundant sequences and the taxonomic assignment was conducted using the RDP classifier.^25^ Software STAMP was used to calculate the *P*-values (ANOVA) for multiple groups/samples within the data sets.^26^ FastTree was used to create phylogenetic trees^27^ for UniFrac distance matrix construction in Mothur.^28^ Bacterial community richness and diversity indices (observed OTUs, Chao 1 estimator and ACE) and rarefaction curves were estimated at 0.97 cutoff. For determination of beta-diversity (OTU based analysis) and clustering (*e.g.* heatmaps), samples were rarefacted to reduce sequence heterogeneity and the UniFrac distance metric was applied to calculate pairwise distances between communities in terms of their evolutionary history.

For the evaluation of the similarity in bacterial community composition among all samples, the relative sequence abundance at class and genus level for each sample was used to calculate pair-wise similarities. All data were transformed by square root calculations and Bray Curtis similarity matrixes were generated using the software Primer v6 (PRIMER-E, Plymouth, UK). Function stress plot draws a Shepard plot, where ordination distances are plotted against community dissimilarities and the fit is shown as a monotone step line. Pyrosequencing data were deposited in the European Nucleotide Archive (ENA) under study accession number of PRJEB5323. To investigate the relationship between water physico-chemical variables and relative sequence abundance at the genus level within biofilm samples, Pearson's correlation coefficients (*r*) were calculated using PASW® Statistics 18 SPSS.

## 3. Results and discussion

### 3.1. Characterization of support media by XPS

**Fig. 1:**
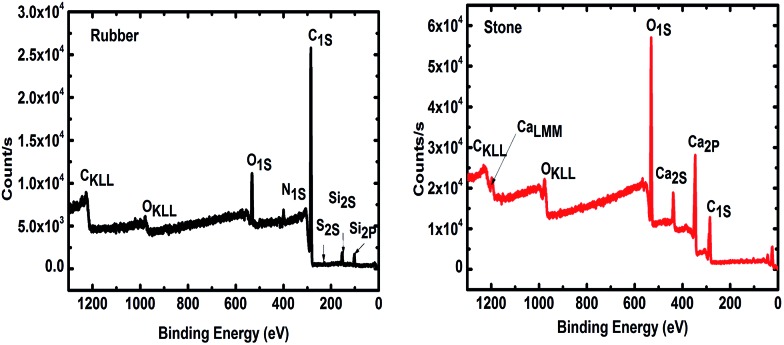
X-ray photoelectron spectrum (XPS) of tire derived rubber (TDR or rubber) and stone media (ST or stone) used as a biofilter for wastewater treatment.

Durable support media and metabolically active biofilms are essential for efficient wastewater treatment bioreactors.^29^ Therefore, it is important to ensure that the elemental composition of the support media is compatible with the microbial community. The intensity of photoelectrons as a function of the binding energy for two support materials (Fig. 1), approve their compatibility with microbial growth. The results show elements C 1s (83.5%), O 1s (9.5%), Si 2p (3.7%), N 1s (2.96%), S 2p (0.30%) and Zn 2p_3_ (0.13%) in both small and large areas of the tested TDR material. Furthermore, the stone media contain C 1s (38%), O 1s (49%), Ca 2p (12%) and Si 2p (1%). This suggests that the main compound of the stone medium is calcium carbonate (CaCO_3_), which is compatible with biofilm development and is known to be quite durable.^30^ The presence of Ca has also been reported to increase the biofilm mass.^31^ It has also been reported that Ca may be used as variable for the improvement of attached growth wastewater treatment systems.^32^ The treatment performance of bioreactors with both support media, stone and TDR, show a similar level of efficiency, with a consistently better performance by TDR media in terms of BOD improvement (Table S2†).

### 3.2. Diversity of the microbial communities

Biofilms were develop on two types of support media (ST and TDR) at three different temperatures, 10, 20 and 30 °C, in laboratory scale aerobic biofilm reactors operated for the duration of 14 days. When TDR was incubated (30 ± 2 °C) with activated sludge showed comparatively higher biofilm development (0.51 g) and physiological activities under aerobic conditions even after 7 weeks.^33^ Khan *et al.* ^34^ found that the starting phase of the stone media FBR was reduced after incubation of the stones with activated sludge for two weeks. In another research study, it was observed that the starting phase of the TF systems packed with different filter media was reduced (36 h at low and 24 h at mesophilic temperature regimes) after inoculation with activated sludge for 14 days for achieving effective treatment of wastewater.^35^

**Fig. 2:**
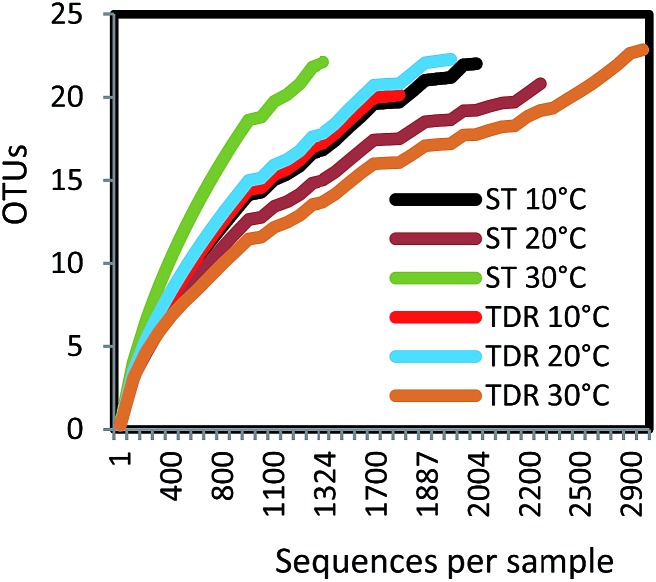
Rarefaction curves of OTUs at 97% of sequence similarity for six biofilm samples from stone (ST) and tire derived rubber (TDR) media.

The analysis of 16S rRNA genes showed a total of 12 142 effective sequence tags recovered from the six samples. The largest number of sequences (2919) was obtained from TDR media biofilm developed at 30 °C, followed by ST at 20 °C (2272), and ST at 10 °C (2004). The smallest number of sequences (1324) was retrieved from ST at 30 °C. The maximum numbers of OTUs (347) were observed in TDR at 20 °C, followed by ST at 10 °C (289). The minimum number of OTUs (256) was obtained from ST at 30 °C (Table S3†). Rarefaction analysis (Fig. 2) showed that all of the curves start to plateau out and coverage figures show satisfactory levels. The calculated Chao 1 indices were 405, 403, 380, 608, 383, and 406 at for ST 10 °C, TDR 10 °C, ST 20 °C, TDR 20 °C, ST 30 °C, and TDR 30 °C, respectively, also demonstrating the highest bacterial diversity for TDR biofilms at 20 °C and lowest for biofilms developed on ST media at 20 °C. On the basis of matrix materials used as biofilm supporting media, the average diversity estimated were 31.60, and 24.00 on TDR and ST, while the average diversity index evaluated on the basis of temperature were 35.31, 23.81 and 23.80 for 10, 20 and 30 °C respectively (Table S3†). The highest dominance of 45.53 was observed on ST at 10 °C; however, Good's coverage estimated for each biofilm sample gives maximum value for TDR at 30 °C.

### 3.3. Similarity analysis of the biofilm samples

**Fig. 3:**
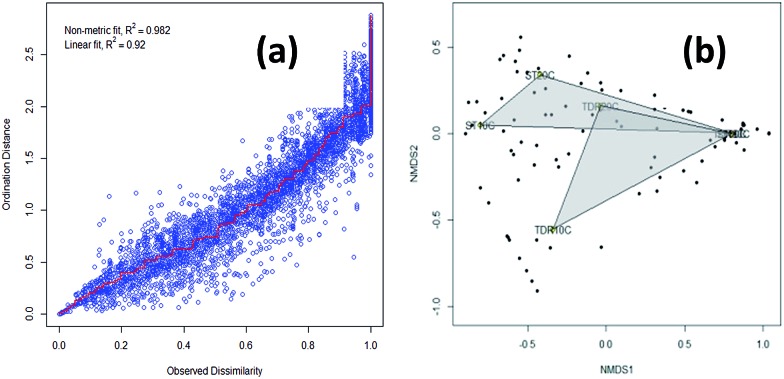
(a) A Shepard plot showing item points (stress) around the regression between distances between each pair of communities against their original dissimilarities and (b) non-metric multidimensional scaling (NMDS) graphs based on Bray–Curtis similarities of the percentage sequence abundance on stone (ST) and tire derived rubber (TDR) media, at 10, 20 and 30 °C showing differences in the bacterial community structure in biofilms developed from real wastewater.

The similarity of the six biofilm samples was evaluated using cluster analysis, and non-metric multidimensional scaling (NMDS). Stress plot values of 0.982 and 0.92 for nonlinear and linear fits respectively, suggested that two sites have uncommon species (Fig. 3a). The plot shows all biofilm communities in the form of clusters obtained from both types of media developed at 10, 20 and 30 °C under aerobic conditions. In Fig. 3b, the NMDS shows a clear separation/difference between TDR and ST biofilms. Different packing media in the reactors provide a suitable environment (moisture, temperature, pH, nutrients, *etc.*) for microbial growth and biofilm formation.^36^ However, a highly significant difference was observed in the biofilm communities developed on these two different types of media (TDR and ST) at 30 °C (Fig. 3b). Although the same wastewater was used as an inoculating agent for biofilm development, the different elemental composition of the media may cause the biofilms to present different diversities.^35^ Ivnitsky *et al.* ^37^ also observed different bacterial compositions in wastewater treating biofilms developed at 20, 25 and 34 °C. Interestingly, the bacterial communities developed at lower temperatures are different than those at mesophilic temperatures.

### 3.4. Core and distinct taxonomic units

**Fig. 4:**
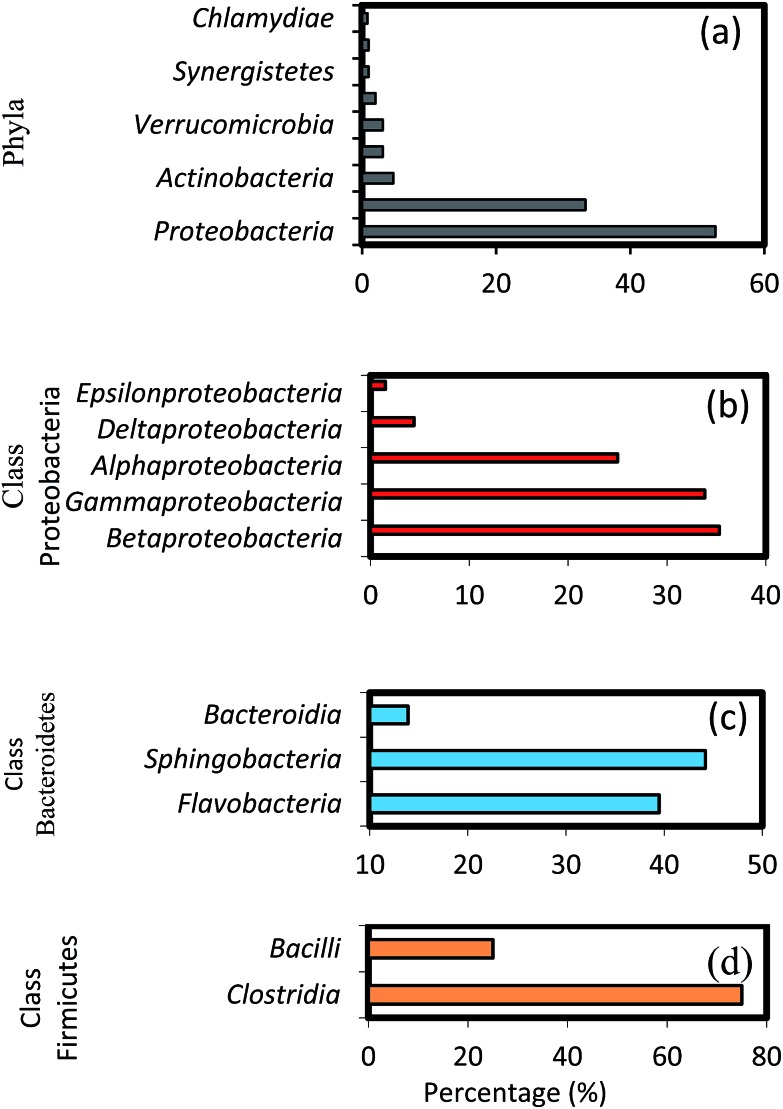
Taxonomic assignments of 16S rRNA gene sequences retrieved from the biofilm samples classified by (a) phyla, (b) classes within major phyla *Proteobacteria*, (c) *Bacteroidetes* and (d) *Firmicutes*.

The results of high-throughput pyrosequencing have revealed interesting microbial community structures in biofilms developed on two distinct support media for municipal wastewater treatment. As shown in Fig. 4a, the phylum *Proteobacteria* was the most abundant in all samples, accounting for 52.71% of total effective bacterial sequences. *Proteobacteria* was found to be the prevalent group in wastewater treating biofilms by other molecular studies,^37^ and in bacterial communities in soil,^38^ sewage^39^ and activated sludge.^40^ The other groups were *Bacteroidetes* (33.33%), *Actinobacteria* (4.65%), *Firmicutes* and *Verrucomicrobia* (3.1%), followed by *Chloroflexi* with average abundance higher than 1%.

These results show some level of agreement with that of pyrosequencing analysis of bacterial diversity in wastewater treatment systems.^16,40^ Within the phylum *Proteobacteria*, the major classes, *i.e.* gamma-, beta-, alpha-, delta- and epsilon-*Proteobacteria* constitute 35.3, 33.82, 25, 4.4 and 1.5%, respectively (Fig. 4b). A high abundance of the gamma-subdivision was found within biofilms; this bacterial group includes most of the known pathogens and opportunistic pathogens, confirming that biofilms are potential reservoirs for such organisms.^41^ Furthermore, the gamma-*Proteobacteria*, preferentially found in active biofilm communities, contain well-known biofilm-forming species, such as *P. aeruginosa*, *V. cholerae*, and *E. coli*, among others.^42^ Biswas *et al.* ^43^ also reported the establishment of a community dominated by gamma-*Proteobacteria* in moving bed biofilm reactor systems for wastewater treatment. Members of these groups have also been shown to auto- and co-aggregate.^44^

It is interesting to note the predominance of beta-*Proteobacteria*; however, this class can attach more easily to surfaces and they dominate the process of biofilm formation in freshwater ecosystems.^45^ To initiate biofilm formation, bacteria need to be able to attach to surfaces or to co-aggregate.^44,46^ This ability might have favoured the proliferation of certain groups of beta-*Proteobacteria*, which were found to dominate biofilm communities in this as well as in earlier studies.^45,47^ Furthermore, studies on the microbial community composition of conventional activated sludge systems indicate that the community is typically dominated by beta-*Proteobacteria*,^48^ followed by alpha-, gamma- and delta-*Proteobacteria*.^40^ These results are also in agreement with the pyrosequencing studies in a fixed-film activated sludge system.^49^ Members of the phylum *Bacteroidetes* occurred predominantly in the biofilm communities after *Proteobacteria*. The relative composition of different genera shown in Fig. 4c (*i.e. Flavobacteria* 39.5%, *Sphingobacteria* 44.2% and *Bacteroida* 13.9%) indicate a comparably low activity of these groups, possibly caused by more favourable growth conditions for these bacteria during early biofilm formation. Interestingly, *Flavobacteria* are known to degrade biopolymers often present in domestic sewage.^8^ Among the *Firmicutes*, the major classes identified were *Bacilli* (75%), and *Clostridia* (25%), as shown in Fig. 4d. *Bacillus subtilis* is also a well-known biofilm forming species,^42^ and the ability of these bacteria to form dormant spores allows them to be resistant to disinfection.^50,51^ Members of other phyla such as *Actinobacteria*, *Verrucomicrobia*, *Cyanobacteria*, *Chloroflexi*, and *Synergistetes* were also found, albeit at relatively low percentages (0.8–4.65%). Some members of the *Chloroflexi* phylum play key roles in submerged membrane bioreactors treating municipal wastewater by eliminating soluble microbial products and cell material largely produced by cellular decay and lysis.^52^ Lebrero *et al.* ^53^ reported that the members of the *Chloroflexi*as dominant bacterial groups in biotrickling filters degrading mixtures of volatile organic compounds such as methyl mercaptan, toluene, alpha-pinene, and hexan.

**Fig. 5:**
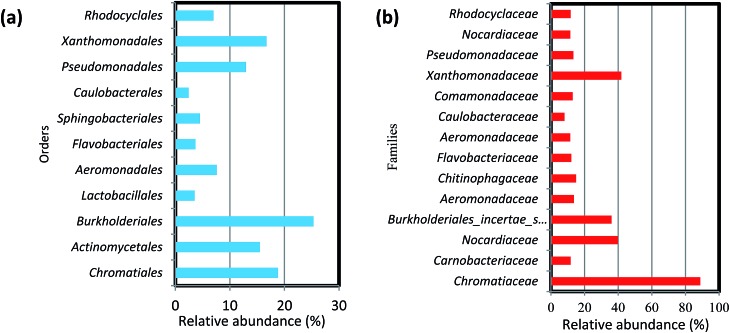
Percentage (%) relative abundance of (a) orders and (b) families in the biofilm samples developed on stone and tyre derived rubber (TDR) media material at 10, 20 and 30 °C from real wastewater in aerobic reactors.

At the order level, the 11 most abundant orders accounted for 50–85% of the community. Among these 11 orders, *Burkholderiales*, *Chromatiales*, *Xanthomonadales*, *Actinomycetes*, *Aeromonadales* were found in all six biofilm samples obtained from both filter media (Fig. 5a). Members of the order *Chromatiales* (37.6 and 17.7%, respectively) were found with greater abundance in the biofilms on ST as compared to TDR at 10 and 20 °C, whereas *Burkholderiales*, *Aeromonadales* and *Actinomycetes* were found in the biofilms developed on TDR at all temperature conditions.

**Fig. 6:**
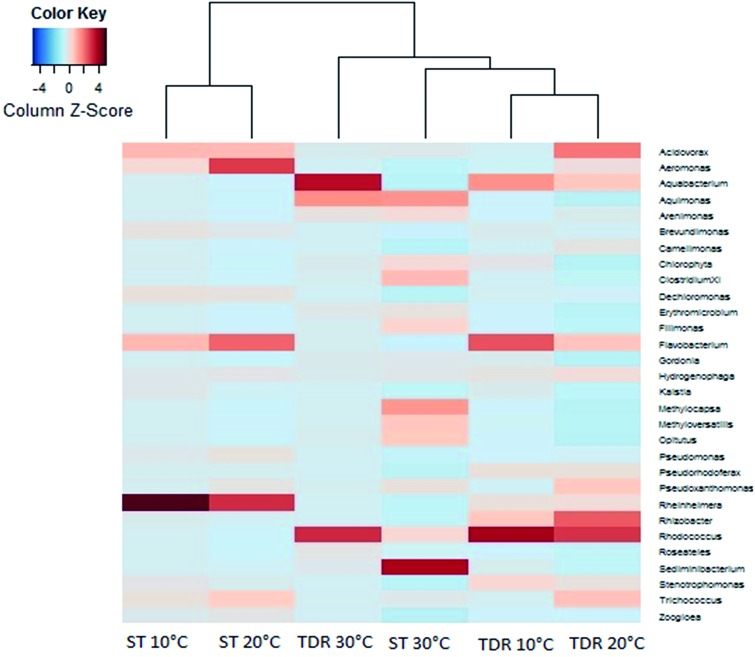
Heatmap showing the most abundant species at the genus level within biofilms retrieved from stone (ST) and tyre derived rubber (TDR) media surfaces developed at 10, 20 and 30 °C in the aerobic reactors.

At the family levels, 14 families were identified with greater relative abundance in all biofilm samples (Fig. 5b). *Chromatiaceae*, *Comamonadaceae*, *Caulobacteraceae*, *Xanthomonadaceae*, *Carnobacteraceae*, and *Pseudomonadaceae* were the families commonly shared by all biofilm samples, whereas *Norcardiaceae*, *Chitinophagaceae* and *Burkholderiaceae* were abundantly found in the biofilms developed on TDR at all three temperatures. However, *Rhodocyclaceae* was noticed in the biofilm samples developed at 10 and 20 °C on both types of media. Some families such as *Rhodocyclaceae*, *Pseudomonadaceae*, *Caulobacteraceae*, and *Sphingomonadaceae* were reported as dominant bacterial families in biofilms of styrene degrading biofilters.^17^ The heatmap (Fig. 6) shows 17 core genera out of 283, including *Rheinheimera*, *Rhodococcus*, *Aquabacterium*, *Trichococcus*, *Acidovorax*, *Flavobacterium*, *Aeromonas*, *Sediminibacterium*, *Hydrogenophaga*, *Aquimonas*, *Brevundimonas*, *Pseudoxanthomonas*, *Rhizobacter*, *Zoogloea*, *Arenimonas*, *Stenotrophomonas*, *Dechloromonas.* These genera were frequently identified in all biofilm samples, but with variable relative abundance (Table S4†). The genus *Rheinheimera* were observed in biofilm samples from both support materials. The species of the genus *Rheinheimera* are able to easily degrade organic matter.^54^ The species in the genus *Zoogloea* are recognized to form zoogloeal matrices,^55^ and are the main mediators for the flocculation of activated sludge processes.^56^ The genus *Dechloromonas* was also observed in all biofilms, but with larger relative abundance (38%) in biofilms retrieved from ST media. *Dechloromonas* has the ability of reducing perchlorate and also reported as phosphate accumulating organisms (PAO's) for the accumulation of phosphorus in the bioreactors.^57,58^ Thus, both test media can be used in reactors for removal of organic wastes, perchlorate, phosphorus, nitrogenous pollutants from wastewater.

**Fig. 7:**
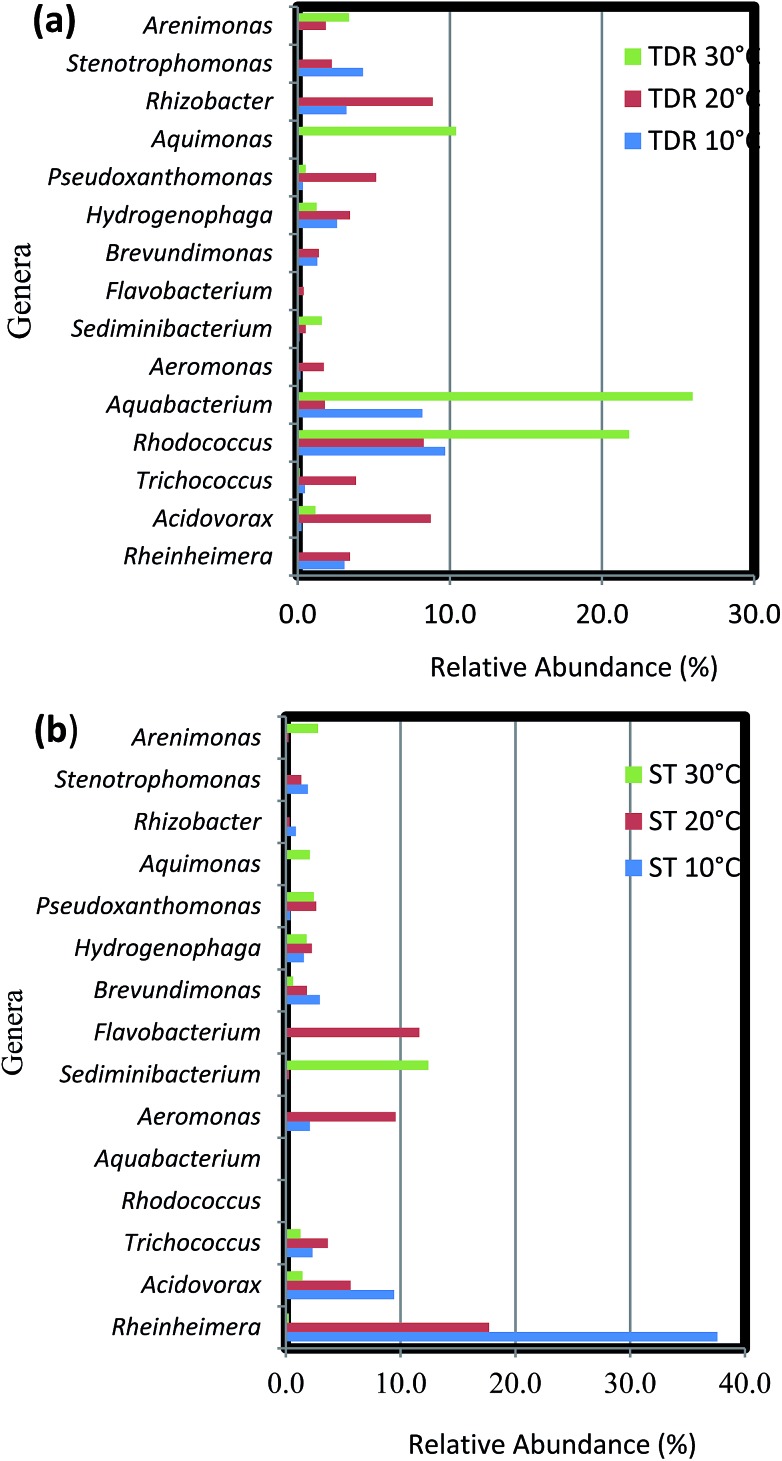
Percentage (%) relative abundance of core genera in the biofilm samples developed on (a) tyre derived rubber (TDR) and (b) stone (ST) media materials at 10, 20 and 30 °C from real wastewater in aerobic reactors.

The biofilm community on TDR medium material at 10 and 20 °C were dominated by phylum *Proteobacteria* with the same proportions of its classes beta-*Proteobacteria* and gamma-*Proteobacteria* (Fig. 7a). However, other classes like alpha-, delta-, and epsilon-*Proteobacteria* were not observed. In the biofilms developed at 20 °C, some *Bacilli* (*Firmicutes*) were also observed. Moreover, on TDR some unique genera such as *Rhodococcus* was found in the biofilms developed at 10, 20 and 30 °C at relative abundances of 23.3, 13.2 and 29.7% respectively (Fig. 7a). The proportions of these genera were significantly higher at 30 °C than at low temperature biofilms (Table S5†). The TDR media support *Rhodococcus* biofilm formation, so it can be considered to be used in the FBRs as a filter media for treatment municipal wastewater. *Rhodococcus* sp. have also been observed in granular activated carbon to remove acrylamide in a laboratory scale trickling filter bioreactors by Zhang and Pierce.^59^ TDR also supported the growth of *Aquabacterium*, *Stenotrophomonas*, *Rhizobacter* and *Erythromicrobium* (Fig. 7a). The genera *Aquabacterium* and *Rhizobacter* were found on ST medium, but their abundance was negligible. Presence of bacteria such as *Erythromicrobium* suggested that it might help to metabolizes iron and manganese within biofilms.^60^ The genus *Erythromicrobium* has also been reported to be able to reduce heavy metals,^61^ suggesting that this bacterium could be of relevance for removing heavy metal ions from polluted industrial wastewaters. While, *Aquabacterium* (19.6%), *Stenotrophomonas* (10.3%), *Rheinheimera* (7.4%) were also found in the biofilm community at 10 °C. Furthermore, *Rhizobacter* (14.4%), *Acidovorax* (13.8%), *Pseudoxanthomonas* (8.3%) were predominantly found in biofilms developed at 20 °C. While, *Aquabacterium* and *Aquimonas* with 35.5 and 14.1% relative abundance was noticed at 30 °C. The genus *Aquimona* is reported to be involved in nitrification in warm springs.^62^

The biofilm samples collected from ST media at 10 and 20 °C had a similar bacterial community composition, with a preponderance of gamma-*Proteobacteria* with genera such as *Pseudoxanthomonas*, *Rheinheimera*, *Stenotrophomonas*, and *Aeromonas* among others (Fig. 7b). It was followed by members of the beta-*Proteobacteria*, with the most dominant genera being *Acidovorax* and *Hydrogenophaga*. While the genera *Brevundimonas* and *Trichococcus* (alpha-*Proteobacteria* and *Firmicutes* respectively) were also observed at considerable abundances in the biofilms. On the other hand, at 30 °C the biofilm community is composed of equal proportions of gamma- and alpha-*Proteobacteria*. Some representative genera of phyla *Firmicutes*, *Bacteroidetes*, *Verrucomicrobia*, *Actinobacteria* and class delta-*Proteobacteria* were also observed in the biofilm community. Most prominent genera at this temperature were *Sediminibacterium*, *Methylocapsa*, *Aquimonas*, *Opitutus*, *Pseudoxanthomonas*, *Rhodococcus etc.* In samples retrieved from ST medium the highest % relative abundance of *Rheinheimera* (58.8 and 25.6%) was noticed in the biofilm samples developed at 10 and 20 °C respectively. While, the *Flavobacterium* (16.8%), *Aeromonas* (13.9%), *Acidovorax* (8.2%) were dominant at 20 °C biofilms. However, the biofilm developed at 30 °C indicated large % relative abundance of *Sediminibacterium* (35.2%), *Methyloversatilus* (10.2%), *Filimonas* (9.1%), *Arenomonas* (7.7%). The difference between bacterial composition at the genus level and its abundance in the samples of the biofilm retrieved at 10 and 30 °C were significant (*P* < 0.001) (Table S6†).

### 3.5. Physico-chemical characteristics of influents and effluents in aerobic growth bioreactors

The physicochemical parameters (BOD, DO and pH) of the influents and effluents at different temperatures from bioreactors were analyzed at three time points according to the approach used in our previous research.^63,64^ These parameters are related to the extent of wastewater treatment and are provided in Table S2.† The results indicate that BOD_5_ of wastewater is strongly influenced by the presence of both heterotrophs and autotrophs in the community. Maximum BOD improvement was observed at 10 and 30 °C in the effluent samples, *i.e.*, 40.9 and 64.2% respectively from the bioreactors packed with TDR media (Table S2†). Dissolved oxygen (DO) is well recognized as a critical process parameter in biological wastewater treatment processes due to its impact on bacterial activity and the high operational costs. The average concentration of DO in the influent supplied to all reactors at 20 °C was 1.95 ± 0.7 mg L^–1^, and 2.06 ± 0.03 mg L^–1^ in all the reactors at 10 and 30 °C, respectively. A considerable increase in the average concentrations was observed in the effluent from all the reactors packed with different types of media. It was also observed that, with the increase in temperature from 10 to 30 °C, the oxygen concentrations were increased in the effluents from reactors. The pH values were near neutral (7.30 ± 0.2) in the wastewater samples used in all the reactors for biofilm development, with a slight reduction at 10 °C and small increase at 20 and 30 °C in reactors with TDR media.

As shown in Table 1, prevailing temperature conditions and OTUs recovered on ST media were strongly positively correlated with each other (*P* < 0.01). The temperature has also a positive correlation (*P* < 0.05) with BOD improvement in the TDR reactors wastewater. The levels of pH were also found to be significantly correlated with BOD improvement (*P* < 0.05) in TDR media reactors. An ideal pH can enhance biofilm growth by increasing exopolysaccharide (EPS) synthesis.^65^ All other parameters have shown non-significant correlation (*P* > 0.05) with each other and also with OTUs and inverse Simpson's index in case of both media reactors (Table 1).

**Table 1:** Pearson correlation coefficient (*r*) for wastewater physico-chemical factors and number of OTUs observed (after 3% cutoff) on stone (ST) and tyre derived rubber (TDR) media^*a*^

	Biofilms							
	Stone (ST)				Tyre-derived rubber (TDR)			
	OTUs	Invisimpson	BOD	DO	OTUs	Invisimpson	BOD	DO
BOD_5_	0.277 (NS)	0.817 (NS)			–0.651 (NS)	–0.988 (NS)		
DO	0.382 (NS)	0.302 (NS)	0.058 (NS)		–0.017 (NS)	–0.0321 (NS)	0.012 (NS)	
pH	0.724 (NS)	–0.021 (NS)	–0.594 (NS)	0.947 (NS)	–0.632 (NS)	–0.993 (NS)	0.999*	0.207 (NS)
Temp (°C)	0.852**	0.961 (NS)	0.685*	0.362 (NS)	–0.044 (NS)	–0.0867 (NS)	0.669*	0.241 (NS)

^*a*^Key: *n* = 9, *p* < 0.01**, *p* < 0.05*, NS = *p* > 0.05; a two tail test was used.

The use of high-throughput pyrosequencing has revealed promising results on the study of diversity and composition of bacterial communities on two different types of packing media developed at 10, 20 and 30 °C in the aerobic biofilm reactors for real-wastewater characteristics (pH, BOD and DO), operational parameters (temperature) and packing media could independently explain the variation in bacterial communities. But interactions among these components seemed to have less influence than did individual components and were overall only observed between operational parameter such as temperature and number of species (OTUs). Certain genera of bacterial populations, including *Rheinheimera*, *Acidovorax*, *Brevundimonas*, *Zoogloea etc.*, appear in all the biofilms studied, and could be considered core genera. In addition to these, some genera were observed with high proportions on certain biofilm support media, such as *Rhodococcus* and *Erythromicrobium*, on TDR, potentially resulting in highly efficient microbial ecology cultivated due to the chemical composition of TDR, for improved BOD removal under common environmental conditions. The capabilities of such microbial population in highly efficient wastewater treatment seem to be enormous, by means of synchronizing support media and environmental condition. These findings can form the basis for reviewing the role of microbial diversity in the biofilm reactors with various packing material and operating temperatures for efficient and cost-effective wastewater treatment. This can assist the selection of suitable support media type for desired biofilm development in fixed biofilm systems at various geographical locations.

## Conflict of interest

The authors declare no competing financial interest.
