# Supplementary material for: Effect of the chemical composition of filter media on the microbial community in wastewater biofilms at different temperatures
Source: RSC Adv. 2016 Oct 26;6(106):104345–53. doi: 10.1039/c6ra21040f (PMC5154295; doi:10.1039/c6ra21040f)
Supplement: Supplementary file 1 [file RA-006-C6RA21040F-s001.pdf]

Electronic Supplementary Material (ESI) for RSC Advances.
This journal is © The Royal Society of Chemistry 2016

Table S1 Barcodes used for different biofilm samples

Description of biofilm samples	Barcode Sequence
Stone 10°C	AAAACACA
Tyre- derived rubber10°C	AAAACGAC
Stone 20°C	AAAACGAG
Tyre -derived rubber 20°C	AAAACACC
Stone 30°C	AAAACGCC
Tyre- derived rubber 30°C	AAAACGCG

Table S2 Physico-chemical properties of the influent and effluent from aerobic reactors at different temperature conditions for stone (ST) and tire derived rubber (TDR) media

Temperature (°C)	Parameters analyzed		Media material	
			ST	TDR
10	BOD (mg/L)	Influent	320.83 ±13.15	320.83±13.15
		Effluent	208.91±09.5	184.87±13.8
	DO (mg/L)	Influent	2.06±0.03	2.06±0.03
		Effluent	4.83±1.0	5.9±0.7
	pH	Influent	7.30±0.2	7.30±0.2
		Effluent	7.24±1.02	7.04±0.8
20	BOD (mg/L)	Influent	378.09±26.9	378.09±26.9
		Effluent	278.66±10.12	246.16±7.67
	DO (mg/L)	Influent	1.95±0.7	1.95±0.7
		Effluent	5.37±1.1	5.95±0.8
	pH	Influent	7.86±0.3	7.86±0.3
		Effluent	7.47±0.6	7.63±0.8
30	BOD (mg/L)	Influent	320.83±13.15	320.83±13.15
		Effluent	181.77±10.09	117.2±22.63
	DO (mg/L)	Influent	2.06±0.03	2.06±0.03
		Effluent	5.84±1.0	6.86±1.02
	pH	Influent	7.30±±0.2	7.30±±0.2
		Effluent	7.03±0.4	6.76±0.21

Key = ± Standard Deviation

Table S3 MOTHUR diversity indices of bacterial communities in twelve aerobic biofilm samples developed on stone (ST) and tire derived rubber (TDR) media

	Nseqs	Sobs	Chao	Chao_Lci	Chao_Hci	lci	Hci	Ace	Ace_Lci	Ace_Hci	Lci	Hci	Invsimpson	Lci	Hci	Coverage
ST10°C	2004	289	404.9565	362.78	471.2433	29.82329	46.87182	464.4569	410.2067	542.9886	38.36069	55.53034	8.620901	0.598613	0.744912	0.936627
TDR 10°C	1714	270	402.2553	351.0448	485.8247	36.21131	59.09249	485.2294	434.3552	551.851	35.97345	47.10859	42.74893	2.838531	3.494899	0.934656
ST20°C	2272	257	379.9362	331.8071	459.0302	34.03236	55.92792	476.9426	424.696	545.4668	36.9439	48.45396	17.84281	0.986054	1.168737	0.952465
TDR 20°C	1909	347	608.0169	521.3074	737.8604	61.31292	91.81315	823.0073	734.5902	931.5942	62.52035	76.78251	42.70273	2.638934	3.197896	0.907805
ST 30°C	1324	256	383.5532	333.8942	464.8708	35.11423	57.50021	395.6414	349.7696	463.9533	32.4362	48.30385	45.5353	4.121054	5.538898	0.916918
TDR 30°C	2919	266	406.0217	351.999	493.9804	38.19983	62.19614	487.4798	435.1462	556.0053	37.00545	48.45488	9.358689	0.415459	0.475116	0.960946

Key: nseqs: number of reads analysed, sobs: species observed at the cut-off value (no of observed OTUs), ace: abundance-based coverage estimator, chao: abundance-based estimator Chao1, Coverage: Good’s coverage. lci and hci: lower and higher confidence interval, respectively.

Table S4 The 16 genera frequently identified in all biofilm samples from stone (ST) and tire derived rubber (TDR) media

**Genus**	**Original Abundance**	
	**Stone (ST) biofilm**	**Tyre derived rubber (TDR) biofilm**
Reheinmera	1019	99
Rhodococcus	26	840
Aquabacterium	4	830
Trichococcus	130	31
Acidovorax	296	167
Flavobacterium	242	43
Aeromonas	235	29
Sediminibacterium	143	52
Hydrogenophaga	94	123
Aquimonas	48	293
Brevundimonas	96	40
Pseudoxanthomonas	89	97
Rhizobacter	22	183
Zoogloea	19	13
Arenimonas	36	117
Dechloromonas	38	19
Stenotrophomonas	62	99

Table S5 *P*-values between biofilm samples developed on tyre derived rubber (TDR) media at 10 and 30°C

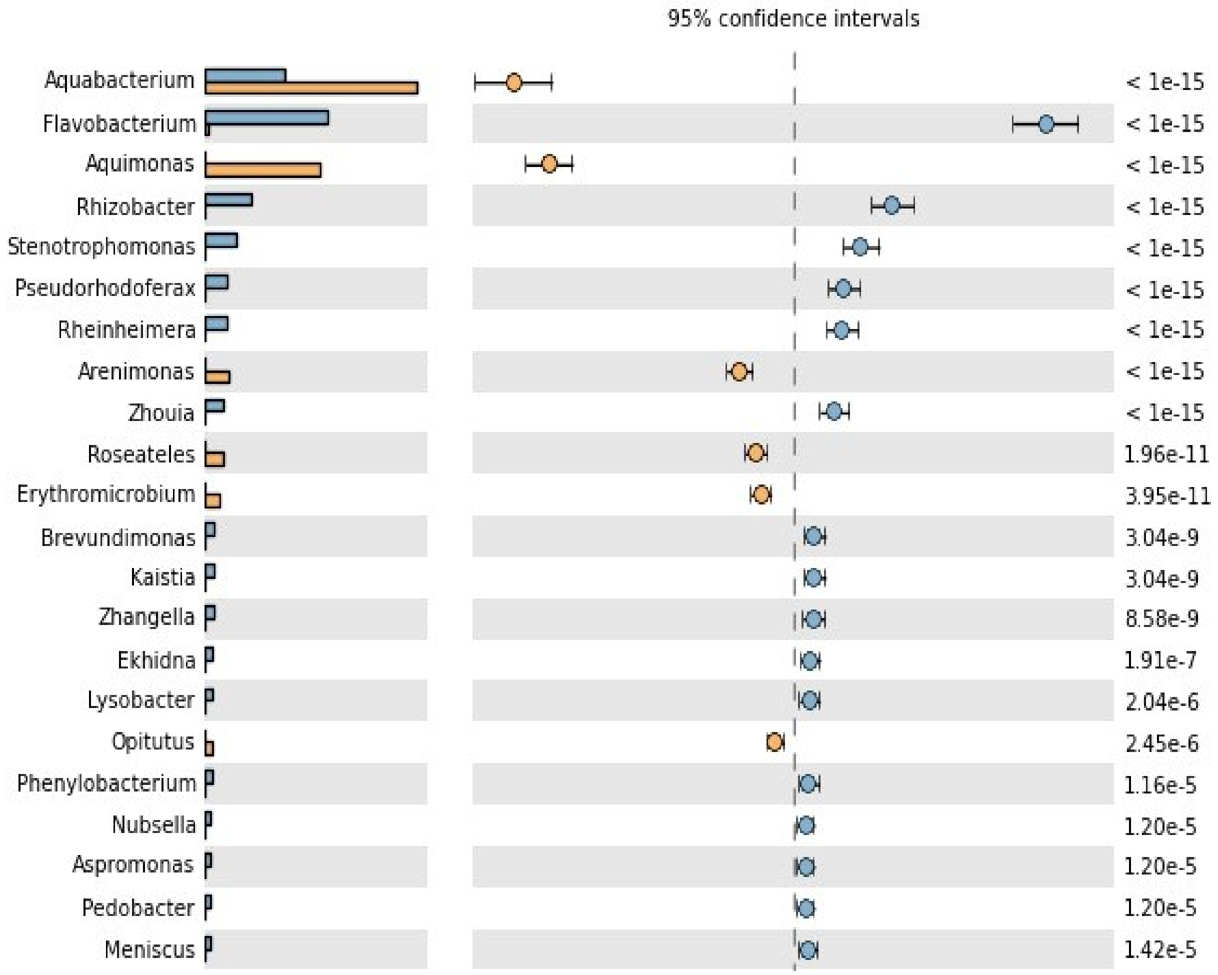

Key: Blue color bars indicating *P*-values between biofilm samples developed at10°C, Orange color bars indicating *P*-values between biofilm samples developed at 30°C.

Table S6 *P*-values between biofilm samples developed on stone (ST) media at 10 and 30°C

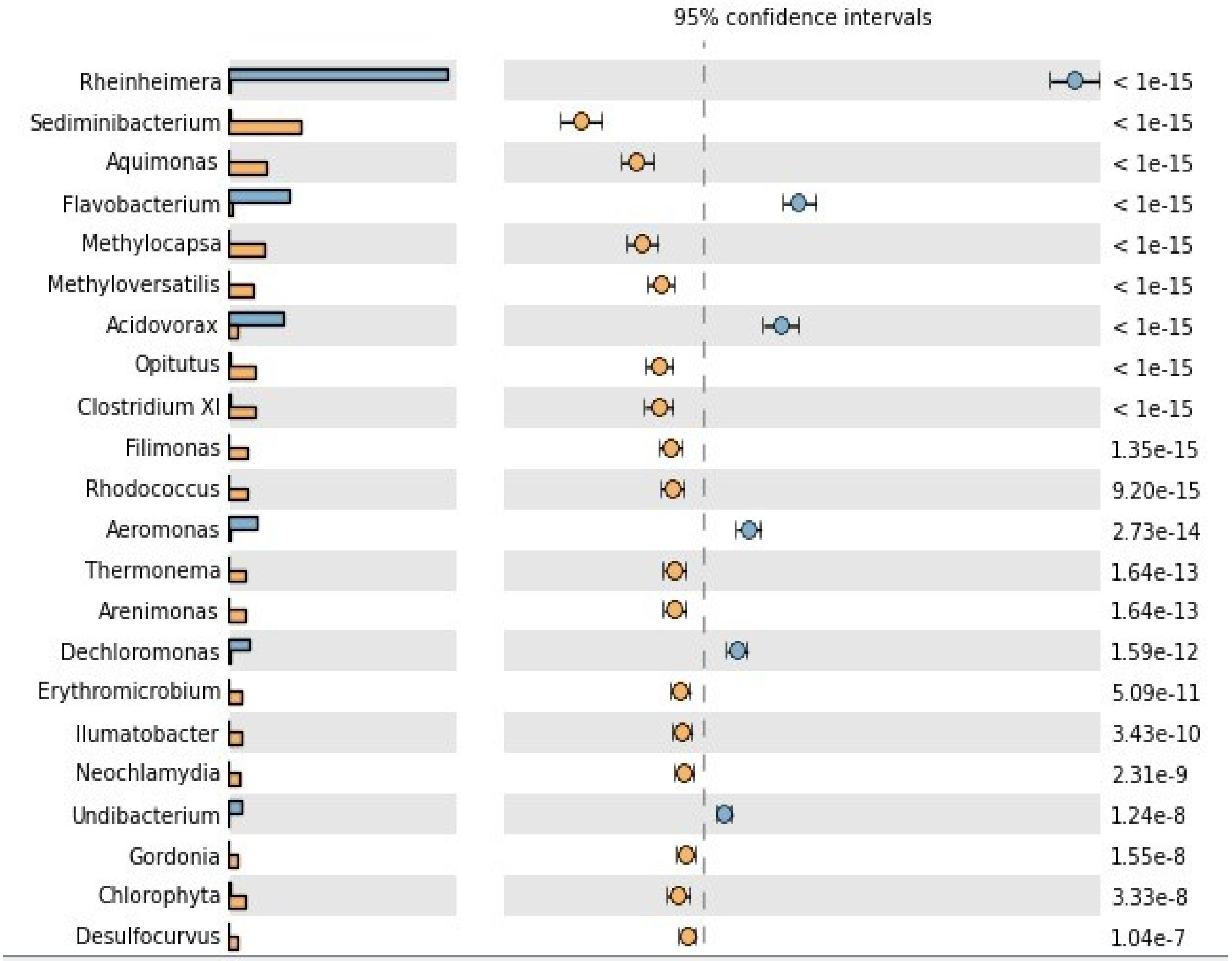

Key: Blue color bars indicate *P*-values between biofilm samples developed at10°C, Orange color bars indicate *P*-values between biofilm samples developed at 30°C.
